# Hyperspectral Monitoring of Powdery Mildew Disease Severity in Wheat Based on Machine Learning

**DOI:** 10.3389/fpls.2022.828454

**Published:** 2022-03-21

**Authors:** Zi-Heng Feng, Lu-Yuan Wang, Zhe-Qing Yang, Yan-Yan Zhang, Xiao Li, Li Song, Li He, Jian-Zhao Duan, Wei Feng

**Affiliations:** ^1^State Key Laboratory of Wheat and Maize Crop Science, CIMMYT-China Wheat and Maize Joint Research Center, Henan Agricultural University, Zhengzhou, China; ^2^Information and Management Science College, Henan Agricultural University, Zhengzhou, China; ^3^College of Science, Henan Agricultural University, Zhengzhou, China

**Keywords:** wheat powdery mildew, spectral transformation, feature band selection, machine learning, remote sensing monitoring

## Abstract

Powdery mildew has a negative impact on wheat growth and restricts yield formation. Therefore, accurate monitoring of the disease is of great significance for the prevention and control of powdery mildew to protect world food security. The canopy spectral reflectance was obtained using a ground feature hyperspectrometer during the flowering and filling periods of wheat, and then the Savitzky–Golay method was used to smooth the measured spectral data, and as original reflectivity (OR). Firstly, the OR was spectrally transformed using the mean centralization (MC), multivariate scattering correction (MSC), and standard normal variate transform (SNV) methods. Secondly, the feature bands of above four transformed spectral data were extracted through a combination of the Competitive Adaptive Reweighted Sampling (CARS) and Successive Projections Algorithm (SPA) algorithms. Finally, partial least square regression (PLSR), support vector regression (SVR), and random forest regression (RFR) were used to construct an optimal monitoring model for wheat powdery mildew disease index (mean disease index, mDI). The results showed that after Pearson correlation, two-band optimization combinations and machine learning method modeling comparisons, the comprehensive performance of the MC spectrum data was the best, and it was a better method for pretreating disease spectrum data. The transformed spectral data combined with the CARS–SPA algorithm was able to extract the characteristic bands more effectively. The number of bands screened was more than the number of bands extracted by the OR data, and the band positions were more evenly distributed. In comparison of different machine learning modeling methods, the RFR model performed the best (coefficient of determination, *R*^2^ = 0.741–0.852), while the SVR and PLSR models performed similarly (*R*^2^ = 0.733–0.836). Taken together, the estimation accuracy of spectral data transformation using the MC method combined with the RFR model (MC-RFR) was the highest, the model *R*^2^ was 0.849–0.852, and the root mean square error (RMSE) and the mean absolute error (MAE) ranged from 2.084 to 2.177 and 1.684 to 1.777, respectively. Compared with the OR combined with the RFR model (OR-RFR), the *R*^2^ increased by 14.39%, and the *R*^2^ of RMSE and MAE decreased by 23.9 and 27.87%. Also, the monitoring accuracy of flowering stage is better than that of grain filling stage, which is due to the relative stability of canopy structure in flowering stage. It can be seen that without changing the shape of the spectral curve, and that the use of MC to preprocess spectral data, the use of CARS and SPA algorithms to extract characteristic bands, and the use of RFR modeling methods to enhance the synergy between multiple variables, and the established model (MC-CARS-SPA-RFR) can better extract the covariant relationship between the canopy spectrum and the disease, thereby improving the monitoring accuracy of wheat powdery mildew. The research results of this study provide ideas and methods for realizing high-precision remote sensing monitoring of crop disease status.

## Introduction

In recent years, global climate change has increased the occurrences of multiple diverse crop diseases, which have resulted in both local and large-scale outbreaks with significant impacts on crop production. The Food and Agriculture Organization of the United Nations (FAO) estimates that 20–40% of global crops are lost worldwide due to pests and diseases every year (Zhang et al., 2020a). Wheat powdery mildew is the main disease of wheat. Powdery mildew disease in some wheat fields has caused large reductions in yield or even complete crop losses, which has seriously affected the security of wheat production in the world. The traditional manual method used to investigate wheat disease information is time-consuming and laborious, and it also leads to mechanical damage to the plants. Therefore, it is important to develop methods for rapid and non-destructive wheat disease monitoring.

Plant diseases leads to a general reduction in plant biomass, destruction of leaf structure, and a decrease in chlorophyll and water contents. The changes in the levels of chlorophyll, water, and other biochemical compounds will inevitably show different absorption and reflection characteristics on the plant reflectance spectrum curve, which makes it possible to use remote sensing technology to monitor wheat diseases in real time (Zhang et al., 2019a). In recent years, with the continuous development of remote sensing technology, many researchers have used it to monitor crop diseases, and have solved many of the shortcomings of traditional methods. Generally, different crops, varieties, and diseases show diversity in spectral characteristics, resulting in different waveband reflectivity to disease sensitivity (Feng et al., 2017). The identification and severity estimation of crop diseases can be achieved by using the spectral response position and the changes in reflectance (Shi et al., 2018; Chen et al., 2019; Franceschini et al., 2019). Some studies have used hyperspectral remote sensing technology to investigate the changes in the spectral reflectance of diseased crops, and have successively constructed sensitive spectral indices to monitor disease status, such as the powdery mildew index (PMI; Mahlein et al., 2013), the double green vegetation index (DGND; Feng et al., 2016), and the red edge vegetation stress index (RVSI; Naidu et al., 2009). With the development of computation methods and the continuous application of novel analyses, many researchers are applying some pre-processing methods to reduce the noise of the original spectral reflectance in order to highlight the characteristics of spectral reflectance and improve the accuracy of remote sensing monitoring. At present, the commonly used methods of spectral transformation are first-order derivative, second-order derivative, logarithm, mean centralization (MC), multivariate scattering correction (MSC), envelope removal, and standard normal variate transform (SNV). When identifying grapes with powdery mildew, Pérez-Roncal et al. (2020) found that SNV combined with MC was the best combination of spectral pretreatment. Kamlesh et al. (2019) estimated the chlorophyll content of African oil palm (*Elaeis guineensis* Jacq.) suffering from Orange Spotting, and found that the model had the highest prediction accuracy using the MSC data set. Kong et al. (2021) found that when monitoring the chlorophyll content and net photosynthetic efficiency of *Brassica napus* affected by haze, MSC is the most optimized spectral pretreatment scheme. Izzuddin et al. (2018) studied that continuous removal (CR) method and showed that it has good value for early disease monitoring in oil palm with basal stem rot (BSR). Thus, it is obvious that the particular spectral transformation method used is of great significance in remote sensing monitoring, but that the appropriate spectral data transformation methods used for different crop types and diseases are not consistent.

Recently, the combination of remote sensing parameters representing different functional traits of plants and machine learning algorithms have been successfully applied to the monitoring of symptoms in plants infected by pathogens (Zarco-Tejada et al., 2018; Poblete et al., 2020). Researchers have developed a variety of machine learning model algorithms that are now widely used in different crop disease monitoring situations (Ashourloo et al., 2016; Ghosal et al., 2018). Regardless of whether the input variable is full band or feature band, machine learning algorithms can perform well in disease classification or regression. Recent studies have shown that feature band selection methods can significantly improve the efficiency of machine learning models without severely sacrificing prediction performance (Huang et al., 2019a; Moghaddam et al., 2020). However, some studies have found that the performance of machine learning algorithms may be significantly affected by the type of input variables or the number of spectral features (Hu et al., 2019; Maimaitijiang et al., 2019), especially in the early detection of rice blast by wavelet analysis combined with the SVM algorithm (Tian et al., 2021). At the same time, Convolutional Neural Networks (CNNs) has been increasingly incorporated in plant phenotyping concepts, successful applied in modeling complicated systems, owing to their ability of distinguishing patterns and extracting regularities from data, such as variety identification in seeds and leaves (Nasiri et al., 2021; Taheri-Garavand et al., 2021a). Therefore, it is particularly important to select the appropriate feature band selection method and machine learning model for crop disease monitoring. In the remote sensing monitoring of wheat powdery mildew, the main problems are: (1) the difficulty encountered in observing the condition of the lower parts of the plants due to the high population density, the diverse canopy structure, and the poor light transmittance, and (2) wheat powdery mildew disease presents a bottom-up pattern of infection. The upper leaf spectral information contributes the most to the canopy spectrum, which leads to a decrease in the sensitivity of the canopy mixed spectrum to wheat powdery mildew. It is necessary to explore the use of pretreatment methods that do not change the spectral curve to enhance the spectral characteristics combined with machine learning model algorithm modeling to reduce the influence of the canopy structure on the estimation of disease severity. At present, there are no studies reporting on detailed research in this area.

Relevant studies have shown that spectral pretreatments such as MC, MSC, logarithmic, first derivative, second derivative, CR, and SNV play an important role and have a reference value for the next step of monitoring modeling work. Although some spectral transformation methods can enhance the spectral features, they also change the shape of the original spectral curve, resulting in the loss of some original features when enhancing certain spectral features. The spectral transformation method used in this study further explored the advantages and values of different spectral transformation methods without changing the shape of the spectrum curve. Therefore, in this study, the research subject was wheat powdery mildew disease, the ground feature hyperspectrometer was used to obtain spectral data combined with ground survey disease data, and used pretreatment methods such as MC, MSC, and SNV. The Competitive Adaptive Reweighted Sampling (CARS) and Successive Projections Algorithm (SPA) algorithms were combined for feature band extraction, and the partial least squares regression (PLSR), support vector regression (SVR), and random forest regression (RFR) algorithms were further used to monitor the incidence of wheat powdery mildew. The results are expected to provide a technical basis for the rapid and large-scale monitoring of wheat powdery mildew, which is of great significance for the precise prevention and control of wheat diseases, improving the efficiency of pesticides, and ensuring food safety and security.

## Materials and Methods

### Experimental Design

Experimental Design (EXP. 1): The test was carried out at the Zhengzhou base (34°51′N, 113°35′E) in the Science and Education Demonstration Park of Henan Agricultural University during the wheat growing season of 2020–2021. The tested varieties were the powdery mildew susceptible varieties “Aikang 58” and “Yumai 49–198.” The first crop was corn, and the straw was crushed and returned to the field after harvest. The soil was loam, and the 0–30 cm soil layer contained 0.99–1.18 g kg^−1^ total nitrogen, 0.023–0.034 g kg^−1^ available phosphorus, 0.114 ~ 0.116 g kg^−1^ available potassium, and the organic matter content ranged from 11.4 to 15.3 g kg^−1^. This test used more water and more nitrogen fertilizer in order to create favorable conditions for powdery mildew disease development. The nitrogen application amount was 270 kg.hm^−2^, and the irrigation amount during the wintering period and jointing stage was 600 and 900 m^3^.hm^−2^, respectively. With the continuous rise of temperature in spring, powdery mildew was inoculated at the jointing stage, and the wheat showed obvious symptoms from the booting stage. The canopy spectrum was detected and sampled at the flowering stage and filling stage. Other field management was the same as that of local wheat conventional measures.

Experimental Design 2 (EXP. 2): Simultaneously with experiment 1, experiment 2 was a variety comparison experimental field including high-sensitivity types (“Yanzhan 4110,” “Nongmai 18,” “Zhoumai 27,” and “Jinfeng 205”) and medium-sensitivity types (“Zhengmai 1342,” “Xumai 318,” “Bainong 207,” and “Xinmai 26”). The amount of nitrogen applied was 225 kg hm^−2^, and the irrigation amount was 675 m^3^ hm^−2^ during the wintering period and the jointing period. This test site was close to fences and pig farms, and the terrain was low-lying. Due to the terrain, air humidity, rainfall, and disease incidence in previous years, the wheat growth environment was suitable for the development and spread of wheat powdery mildew. Without field inoculation, the disease was natural and the disease condition was more severe. Other field management practices were the same as for EXP. 1.

### Field Data Collection

#### Investigation of Disease Index and Leaf Area Index

In this study, the prevalence of wheat powdery mildew infection was investigated manually at the flowering and filling stages of wheat. In this study, the mean disease index (mDI) was used to reduce the influence of canopy structure diversity on the estimation of disease severity (Feng et al., 2016). In experimental design 1 and experimental design 2, we collected 77 and 37 samples respectively, surveyed approximately 0.2 m^2^ at each point in the experimental area, and selected 20 representative wheat plants and brought them back to the laboratory. In the laboratory, all fully expanded green leaves were separated, and the leaf area (LA) was measured using the grid method (Feng et al., 2019). Since there is a strong correlation between the area of the fresh leaves and their dry weight (DW), the leaves were placed in an oven at 105°C for 30 min to inactivate endogenous enzymes, and then heated at 80°C to a constant weight. Finally, the DW of the leaves from 20 plants was obtained, the remaining leaves were weighed, and the leaf area index (LAI) was then calculated using formula (1). The disease index was investigated in strict accordance with the technical specifications for crop disease monitoring. The ratio of the LA covered by the mycelium layer (lesions) on the diseased leaves to the total LA was expressed by a scoring method with 8 levels; 1, 5, 10, 20, 40, 60, 80, and 100%. The grid method was then used to calculate the ratio of disease lesion area to total LA. This was done by covering the leaves with a grid and recording the total number of squares and the number of squares with disease lesions. In order to facilitate the calculation of the disease score, an approximate value was taken for the disease between the grades. If disease lesions were present but the severity was <1%, it was recorded as 1%. The average severity of leaf disease was calculated using formula (2).


(1) $$LAI=\frac{\left(\mathrm{LA}\times \frac{DW_{1}+DW_{2}}{DW_{1}}\right)}{S}$$

$DW_{1}$ and $DW_{2}$ are the dry weights of the leaves and the remaining leaves of 20 plants, respectively, and *S* is the sampling area of each plot. LAI is a dimensionless indicator to characterize the vigorous degree of vegetation.


(2) $$D=\frac{\sum \left(D_{i}\times L_{i}\right)}{L}\times 100$$

In the above formula, *D* is the average disease score to reflect the severity of the disease, which is expressed as a percentage (%); *D*_i_ is each disease score; *L*_i_ is the number of diseased leaves corresponding to each disease score, and the unit was blade; *L* is the total number of leaves in the survey, the unit was the blade.

On the basis of the scores of the diseased leaves, the mDI (%) was calculated to represent the average level of disease occurrence (formula 3).


(3) $$mDI=\frac{F\times D}{LAI}\times 100$$

where *F* is the diseased leaf rate, and *D* is the average score of the diseased leaves.

#### Canopy Spectrum Data Measurement

Canopy spectroscopy is carried out at flowering and filling stages, at which time it is less affected by the soil background. The diameter of the ground field of view was 0.44 m. From 10:00 to 14:00 (Beijing local time) when there was either no wind or low wind velocity, a FieldSpec handheld spectrometer (FieldSpec Handheld 2, Analytical Spectral Devices, Boulder, CO, United States) was used to measure the canopy spectrum. The field of view of the spectrometer is 25° in the 325–1,075 nm band, the spectral sampling interval was 1.4 nm, and the spectral resolution was 3.0 nm. A 0.4 m × 0.4 m BaSO4 calibration plate was used to calculate black and baseline reflectance. Ten spectra were recorded at each sampling point as the samples, and the average value was taken as the spectral reflectance of the sampling area.

#### Spectral Preprocessing

Hyperspectral data has the characteristics of complexity and high dimensionality. It not only contains sample information, but also background information and noise (Wei et al., 2019). In order to partially eliminate the non-information spectrum affected by light scattering and noise, the Savitzky–Golay function was first used in Matlab to de-noise the spectrum data, and the smoothed spectrum was recorded as the original spectrum (OR). Following this, the corresponding spectral transformation was performed to extract useful spectral information, which provided an information source for the subsequent development of multivariate models. The spectral transformation methods were mainly MC, MSC, and SNV which were used to eliminate noise and scattering effects and to enhance the spectral characteristics that truly reflect the target information. The above spectral data preprocessing was all carried out using Matlab software (The MathWorks Inc., Natick, MA, United States). The formulas about MC, SNV, and MSC are as follows.


(4) $$R_{i,MC}=R_{i}-\bar{R_{i}}$$

(5) $$R_{i,SNV}=\frac{R_{i,k}-\bar{R_{i}}}{\sqrt{\frac{\sum_{k=1}^{m}{\left(R_{i,k}-\bar{R_{i}}\right)}^{2}}{m-1}}}$$

In the above formulas, $\bar{R_{i}}$ is the average of the i sample spectra; k=1,2,3,…m. k is the number of wavelength points; m=1,2,3…,n . n is the sample size.


(6) $$R_{i}=a_{i}\bar{R}+b_{i}$$

(7) $$R_{i,MSC}=\frac{R_{i}-b_{i}}{a_{i}}$$

In the above formulas, $\bar{R}$ is the average of all sample spectra; The spectra of each sample were subjected to a one-dimensional linear regression against the mean spectrum, with $a_{i}$ and $b_{i}$ being the coefficients of the regression.

### Feature Band Selection Method

The CARS algorithm is a feature band selection method based on Adaptive Reweighted Sampling (ARS) and Darwin’s theory of evolution (Sun et al., 2019). Monte Carlo was used for PLSR cycle modeling analysis, and cross-validation (CV) was used to evaluate the subset. Finally, the variables with large errors were eliminated, and the characteristic variables were selected after multiple sampling cycles.

The SPA is a variable selection algorithm that selects characteristic variables by calculating the size of the projection vector of the remaining variables and the selected variables, which can ensure that the linear relationship between the selected variables is minimized so as to eliminate redundant information between variables and reduce multicollinearity, and achieve the purpose of selecting feature variables (Jia et al., 2019).

The above CARS algorithm and SPA algorithm have their own advantages. Therefore, CARS and SPA were combined to evaluate the importance of feature wavenumbers. Specifically, CARS was first investigated to obtain a group of potential characteristic variables related to mDI. To assess the significance of these survived spectra, SPA was then applied to select the most effective variable subsets from such potential feature wavelengths. It is recommended to adopt the variables containing the most effective information to establish simplified models for rapid analysis. According to previous studies, CARS–SPA algorithm has not yet been used for feature wavelength selection of wheat powdery mildew samples in the visible and near-infrared spectral regions.

### Estimation Models

#### Partial Least Squares Regression

Partial least squares regression is a classic modeling method that includes the characteristics of PCA, canonical correlation analysis, and multiple linear regression analysis. It is often used for remote sensing quantitative analysis (Guo et al., 2021). PLSR transforms the original variables with high data redundancy into a few variables by selecting the optimal latent variables to describe the relationship between the predicted value and the true value.

#### Support Vector Regression

The basic idea of SVR is to use training samples to establish a regression hyperplane and to turn approximate the samples to the hyperplane to minimize the total deviation from the sample point to the plane (Han et al., 2020). The kernel functions commonly used in SVR algorithm include linear kernel function, radial basic function (RBF) kernel function, polynomial kernel function, and sigmoid kernel function. Among these, the RBF kernel function can deal with complex nonlinear problems between independent variables and dependent variables.

#### Random Forest Regression

Random forest regression is a machine learning algorithm based on a classification regression tree (Breiman, 2001). Random forest regression uses the bootstrap resampling method to extract multiple samples from the original sample and model each bootstrap sample into a decision tree. It then combines them into multiple decision trees for prediction, and uses the majority voting method to determine the final classification result of the joint prediction model. The advantage of this method is that the training speed is relatively fast and does not require CV. At the same time, the randomness of sampling and feature selection means that the RFR does not easily fall into overfitting (Lu and He, 2019), and it is therefore widely used in remote sensing monitoring.

A grid-search and five-fold cross-validation techniques were applied to obtain optimal parameters for each method during the model calibration phase. For the PLSR method, the number of principal components (PCs) was determined by the grid search. RBF was used for SVR, and the kernel parameter gamma and regularization parameter were determined by tuning. The number of trees for the RFR method was set at 200, and the max features parameter, which decides how many features each tree in the RFR considers at each split, was determined through the grid search procedure. Data analysis was performed in Matlab (The MathWorks Inc., Natick, MA, United States).

### Model Verification

**Figure 1:**
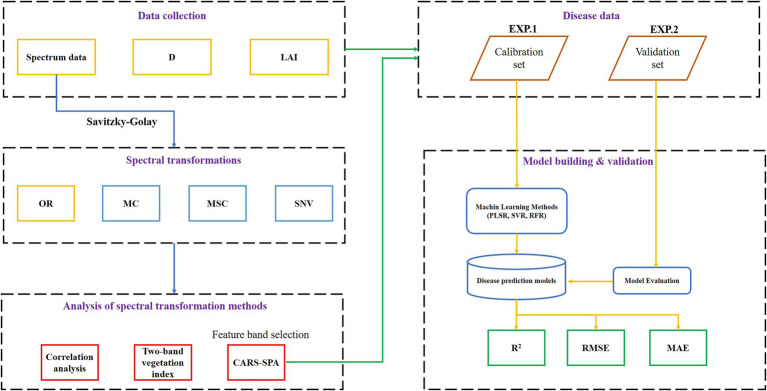
The overall technical workflow of the study.

Taking the characteristic band under spectral transformation as the independent variable and mDI as the dependent variable, a monitoring model of the wheat powdery mildew disease index was constructed based on three modeling algorithms. In order to make the model evaluation result more objective, the data from experiment 1 (EXP.1) was used as the modeling set, and the data from experiment 2 (EXP.2) was the verification set. The workflow from data collection to model building and evaluation is shown in Figure 1. The accuracy of the disease index monitoring model was evaluated by three indicators: the coefficient of determination (*R*^2^), the root mean square error (RMSE), and the mean absolute error (MAE). The closer *R*^2^ is to 1, the lower the RMSE and the lower the MAE, and the higher the accuracy of the monitoring model.


(8) $$R^{2}=\frac{\sum_{i=1}^{n}{\left(x_{i}-\bar{x}\right)}^{2}\times {\left(y_{i}-\bar{y}\right)}^{2}}{\sum_{i=1}^{n}{\left(x_{i}-\bar{x}\right)}^{2}\times \sum_{i=1}^{n}{\left(y_{i}-\bar{y}\right)}^{2}}$$

(9) $$RMSE=\sqrt{\frac{\sum_{i=1}^{n}{\left(y_{i}-x_{i}\right)}^{2}}{n}}$$

(10) $$MAE=\frac{\sum_{i=1}^{n}\left|y_{i}-x_{i}\right|}{n}$$

In the above formulas, $x_{i},\bar{x},y_{i},\bar{y}\;$ are the measured mDI and the average mDI, the predicted mDI and the average mDI, respectively; *n* is the number of samples.

## Results

### Changes in Spectral Characteristics of the Wheat Canopy and Its Correlation With Disease Index

**Figure 2:**
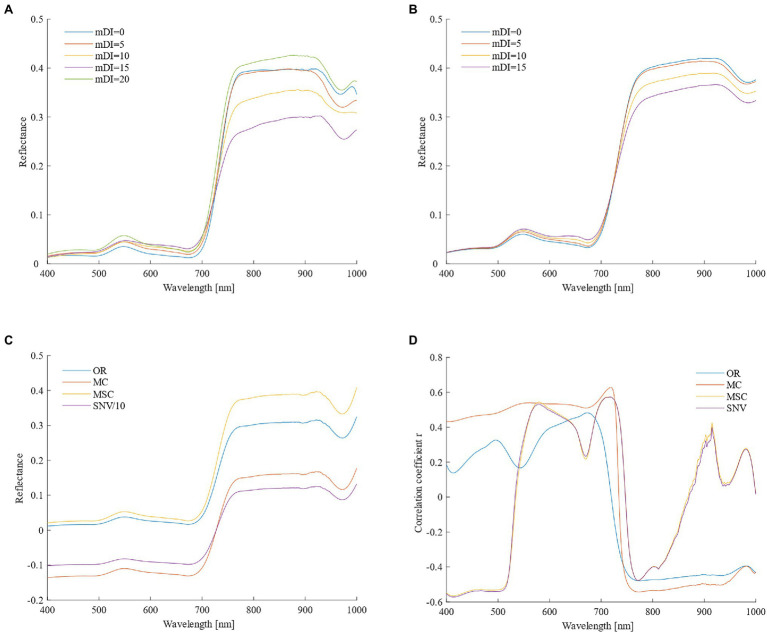
Changes in spectral reflectance of the wheat canopy and correlation with the powdery mildew disease index (**A,B**, Original reflectance of different disease severities in the high-sensitivity and medium-sensitivity types; **C**, spectral reflectance from the four different pretreatment methods; and **D**, correlation coefficients between spectral data and disease indexes).

Figures 2A,B show that as the disease index increased, the original spectral reflectance of the visible light band from 400 to 780 nm gradually increased, and the discrimination was higher. The original spectral reflectance in the near-infrared band of 780–1,000 nm was less discriminating in the spectrum curve when the disease was mild, and the sensitivity was poor. When the disease severity was below moderate, the original spectral reflectance gradually reduced, and when the disease severity was high (mDI = 20), due to the severe damage of the canopy structure, the spectral reflectance increased sharply, even exceeding the spectral reflectance of healthy wheat plants.

Three spectral transformation methods were used for spectral preprocessing, and none of them changed the shape of the spectral curve (Figure 2C). It can be seen from Figure 2D that the Pearson correlation coefficient of the spectrum and mDI after MC pretreatment was significantly improved in most bands, especially the correlation coefficient at 719 nm, which reached 0.628 (*p* < 0.001). The Pearson correlation coefficient curves for the MSC and SNV pretreatment methods were similar, and the correlations in the ranges 400–530 nm, 540–635 nm, 688–740 nm, and 760–1,000 nm were significantly improved compared to the original spectrum, especially the direction of the correlation in the range of 400–530 nm was changed. After comprehensive comparisons of the different preprocessing methods, MC could be considered to be the best preprocessing method.

### Two-Band Combination Optimization Analysis After Spectral Transformation

**Figure 3:**
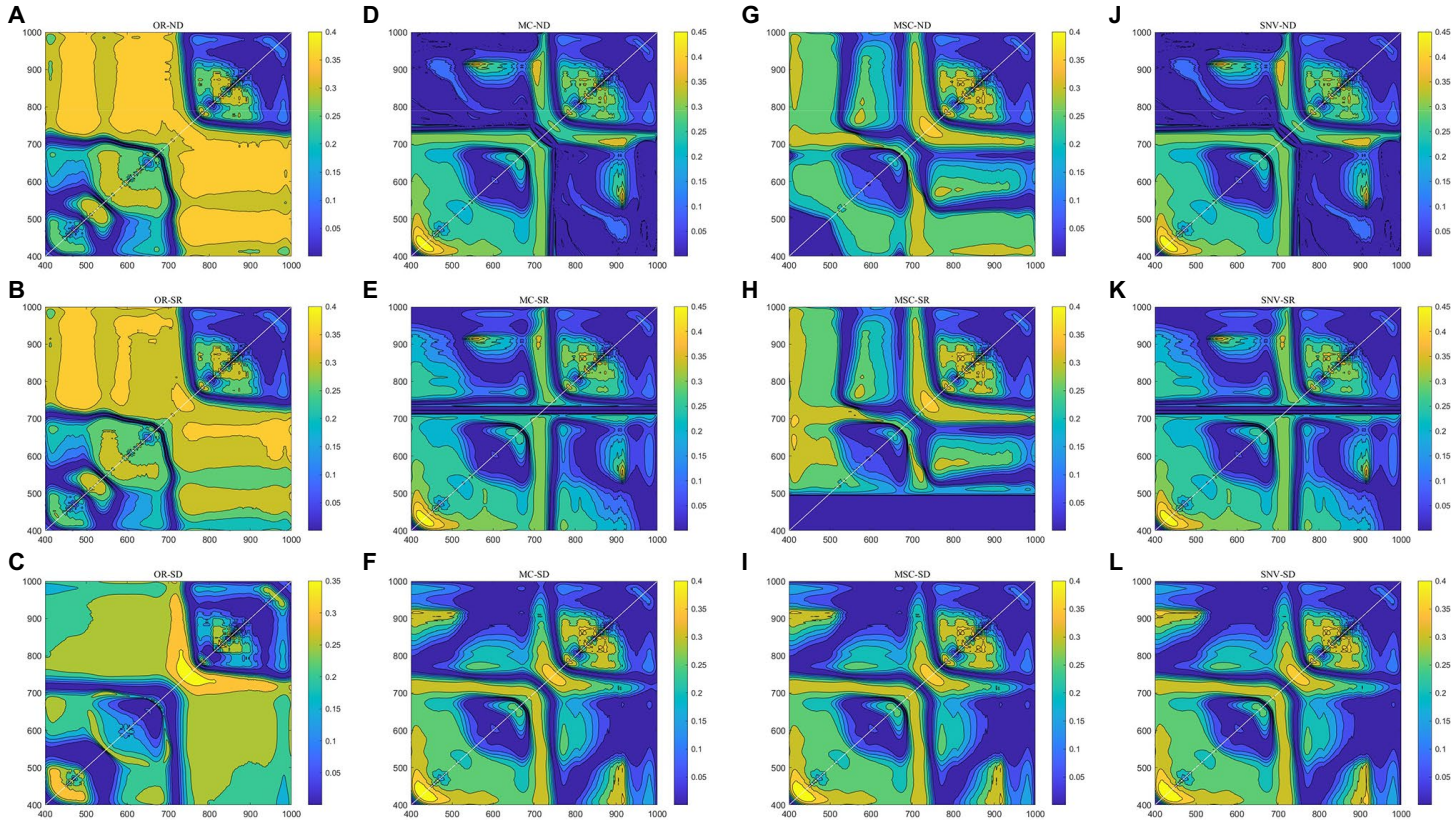
Determination coefficients for optimal two-band combinations with the different spectral variation methods [**A–C** for original reflectivity (OR); **D–F** for mean centralization (MC); **G–I** for multivariate scattering correction (MSC); and **J–L** for standard normal variate transform (SNV)].

Two-band combination optimization analysis was performed on the four kinds of spectral data (Figure 3). On the whole, the bands of the original spectrum are more adaptable and have more two-band combinations with better correlations. Among the three spectral preprocessing methods, there were more combinations of invalid bands with lower correlation. From the perspective of the best combination band, the *R*^2^ of the ND and SR combination of the original spectrum was slightly greater than 0.40, while the SD combination is only 0.378 (Table 1). However, the *R*^2^ of the spectral data after MC and SNV transformation was significantly improved in the ND and SR combination, and the highest *R*^2^ reached 0.48, but the *R*^2^ of the SD combination did not show a significant improvement. In the MSC method, *R*^2^ was only slightly improved in ND, even the SR combination showed a decline, while the *R*^2^ of the SD combination showed a larger increase, with the highest *R*^2^ being 0.447. The performance of the four spectral transformation methods in the optimal combination of two bands can be summarized in the order SNV > MC > MSC > OR.

**Table 1:** Optimized forms of optimal two-band combinations and monitoring performance.

Spectrum transform	Normalized difference vegetation index (ND)		Simple ratio vegetation index (SR)		Simple difference vegetation index (SD)	
	Bands	*R* ^2^	Bands	*R* ^2^	Bands	*R* ^2^
OR	1,000,485	0.401	668,1,000	0.417	750,749	0.378
MC	431,430	0.480	430,432	0.480	750,749	0.378
MSC	786,785	0.409	786,785	0.409	786,785	0.447
SNV	431,430	0.480	430,432	0.480	431,430	0.449

### Optimal Selection of Characteristic Bands

**Figure 4:**
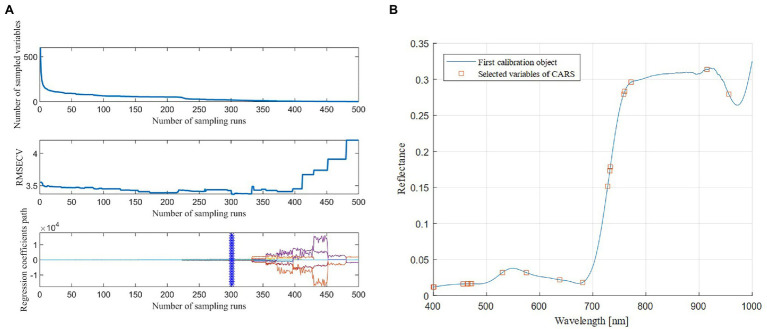
The process of band selection by the Competitive Adaptive Reweighted Sampling (CARS) algorithm **(A)** and the bands selected by the CARS algorithm **(B)**.

**Figure 5:**
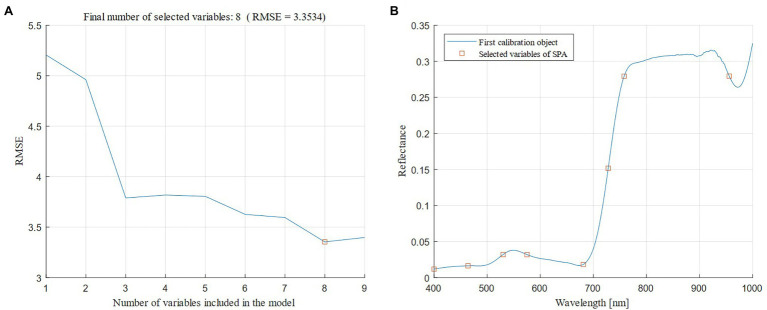
Variation in the root mean square error (RMSE) in the Successive Projections Algorithm (SPA; **A**) and the optimal bands chosen by the SPA algorithm **(B)**.

The combination of CARS and SPA was used to select the characteristic bands in the four types of spectral data transformation. Taking the original spectrum as an example (Figure 4A), as the number of CARS iterations increased, the number of characteristic bands gradually decreased, and the RMSE obtained by 10-fold cross-validation slowly decreased, and then significantly increased. When the number of iterations reached 411, the key information of the spectrum was lost, which led to poor model performance. At 301 iterations, the RMSE reached the minimum value (RMSE = 3.368), and 19 bands were selected (Figure 4B), accounting for 0.316% of the full band. After that, the 19 bands were selected for the second time using the SPA algorithm, the minimum number of bands was two, and the maximum number of bands was 19. Figure 5A showed that as the number of variables continued to increase, the RMSE continued to decrease, and then tended to rise. When the number of variables was eight, the RMSE reached the minimum value (RMSE = 3.353), and eight best bands were selected (Figure 5B).

**Figure 6:**
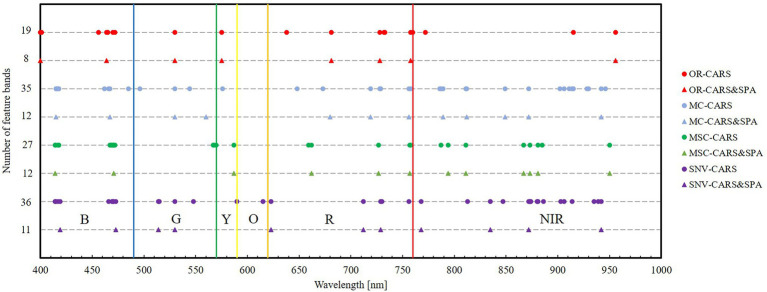
Location and number of feature bands selected by the eight models.

It can be seen from Figure 6 and Table 2 that the pre-processed spectral data was screened using the CARS algorithm, and the number of selected bands was significantly higher than in the original spectrum without transformation, showing the effectiveness of the spectral transformation method. After the second optimization using the SPA algorithm, the number of characteristic bands dropped sharply, some similar bands were eliminated, and the band position distribution under each spectral data type was relatively uniform; in particular, the number of bands was increased in the near-infrared band.

**Table 2:** Results of feature band selection for four different spectral transformation methods.

Spectrum transform	Optimum band selection using SPA on the base of CARS
OR	400,464,530,575,681,728,758,956
MC	415,467,530,560,680,719,756,789,812,849,872,942
MSC	414,471,587,662,727,757,794,811,867,873,881,950
SNV	419,473,514,530,623,712,729,768,835,872,942

### Disease Severity Estimation Models Based on Different Modeling Algorithms

**Figure 7:**
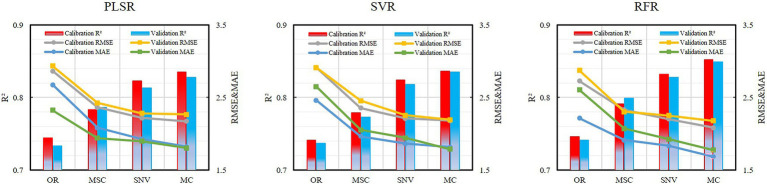
Performance of spectral transformation data for the different models.

The characteristic bands screened by the different preprocessing methods were quite different, and the three machine learning modeling methods, PLSR, SVR, and RFR, were further used to construct the disease index estimation model (Table 3; Figure 7).

**Table 3:** Specific metrics for machine learning models based on different spectral transformation methods.

Spectrum transform	Number of variables	Modeling method	Calibration set			Validation set		
			*R* ^2^	RMSE	MAE	*R* ^2^	RMSE	MAE
OR	8	PLSR	0.744	2.865	2.325	0.733	2.934	2.675
		SVR	0.741	2.913	2.463	0.737	2.912	2.651
		RFR	0.746	2.728	2.215	0.741	2.872	2.604
MSC	12	PLSR	0.783	2.365	1.941	0.786	2.424	2.084
		SVR	0.779	2.354	1.964	0.773	2.456	2.054
		RFR	0.791	2.331	1.912	0.799	2.304	2.073
SNV	11	PLSR	0.823	2.217	1.898	0.813	2.282	1.929
		SVR	0.824	2.211	1.865	0.818	2.254	1.945
		RFR	0.832	2.202	1.835	0.828	2.252	1.924
MC	12	PLSR	0.835	2.173	1.802	0.828	2.268	1.822
		SVR	0.836	2.180	1.817	0.835	2.193	1.788
		RFR	0.852	2.084	1.684	0.849	2.177	1.777

By using the CARS–SPA algorithm on the original spectrum to screen eight characteristic bands as the input of the model, the modeling set and verification set for the different modeling methods were stable at 0.73–0.75, and the RMSE and MAE were also relatively stable. The performances of each model algorithm were not very different. Among them, the RFR model had the highest accuracy. The *R*^2^, RMSE, and MAE of the modeling set were 0.746, 2.728, and 2.215, respectively, and the *R*^2^, RMSE, and MAE of the verification set were 0.741, 2.872, and 2.604, respectively.

**Figure 8:**
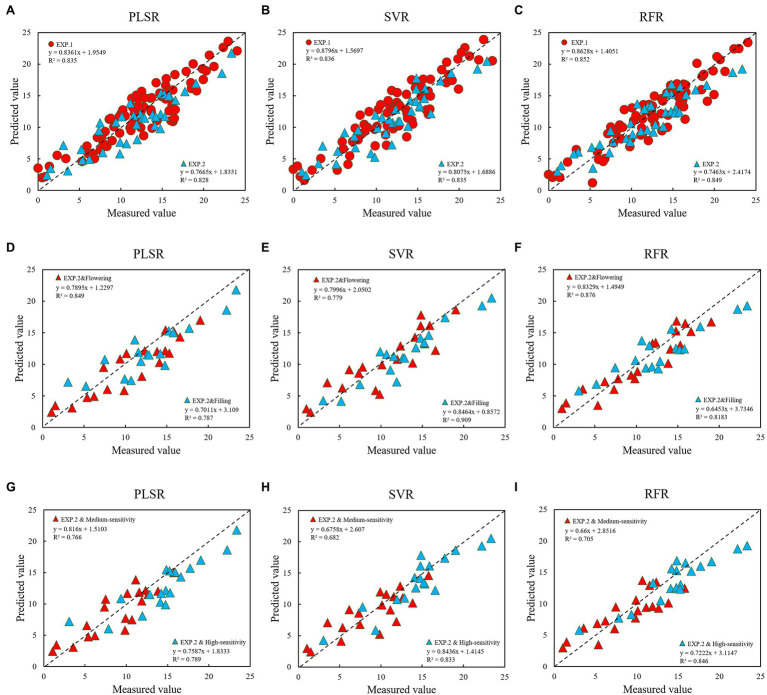
Comparison between the different models using the MC pre-processing method (**A–C** are modeling and testing of the model; **D–F** are the performance of different growth stages in the modeling set, and **G–I** are the performance of high-sensitivity and medium-sensitivity types in the modeling set).

The transformed spectral data performed well in each model, and the results were improved compared with the original spectral model. Of these, the improvement effect of MSC was small. Taken together, the *R*^2^ of the modeling set and the verification set were increased by 5.5 and 6.6%, the RMSE was reduced by 17.1 and 17.6%, and the MAE was decreased by 16.9 and 21.7%, respectively. However, the effects of SNV and MC were greatly improved. For both the modeling set and the verification set, *R*^2^ was increased by at least 10%, and RMSE and MAE were both reduced by over 20%.In a comprehensive comparison, the 12 characteristic bands selected by MC combined with the CARS–SPA algorithm performed best in each modeling method, followed by SNV, MSC, and OR, which indicates that the appropriate spectral data transformation prediction was selected to enhance the spectral response characteristics of the target object, so that the accuracy of the inversion model was further improved. Figures 8A–C show the comparison of the three modeling methods based on MC preprocessing. The best modeling results were obtained after using the best pre-treatment method MC, with the PLSR and RFR models generating better performance at flowering than at filling, while the SVR showed diametrically opposed results, as seen in the scatter plots from the growth stages (Figures 8D–F). However, the highly sensitive types were better monitored than the moderately sensitive types, and overestimation is still a problem to be solved at lower disease levels (mDI < 5; Figures 8G–I). In summary, the RFR model performed best, followed by SVR and PLSR. The SVR model was more stable than the PLSR model. SVR model was more stable than the PLSR model.

### Importance Analysis of the Input Variables of the Optimized RFR Model

**Figure 9:**
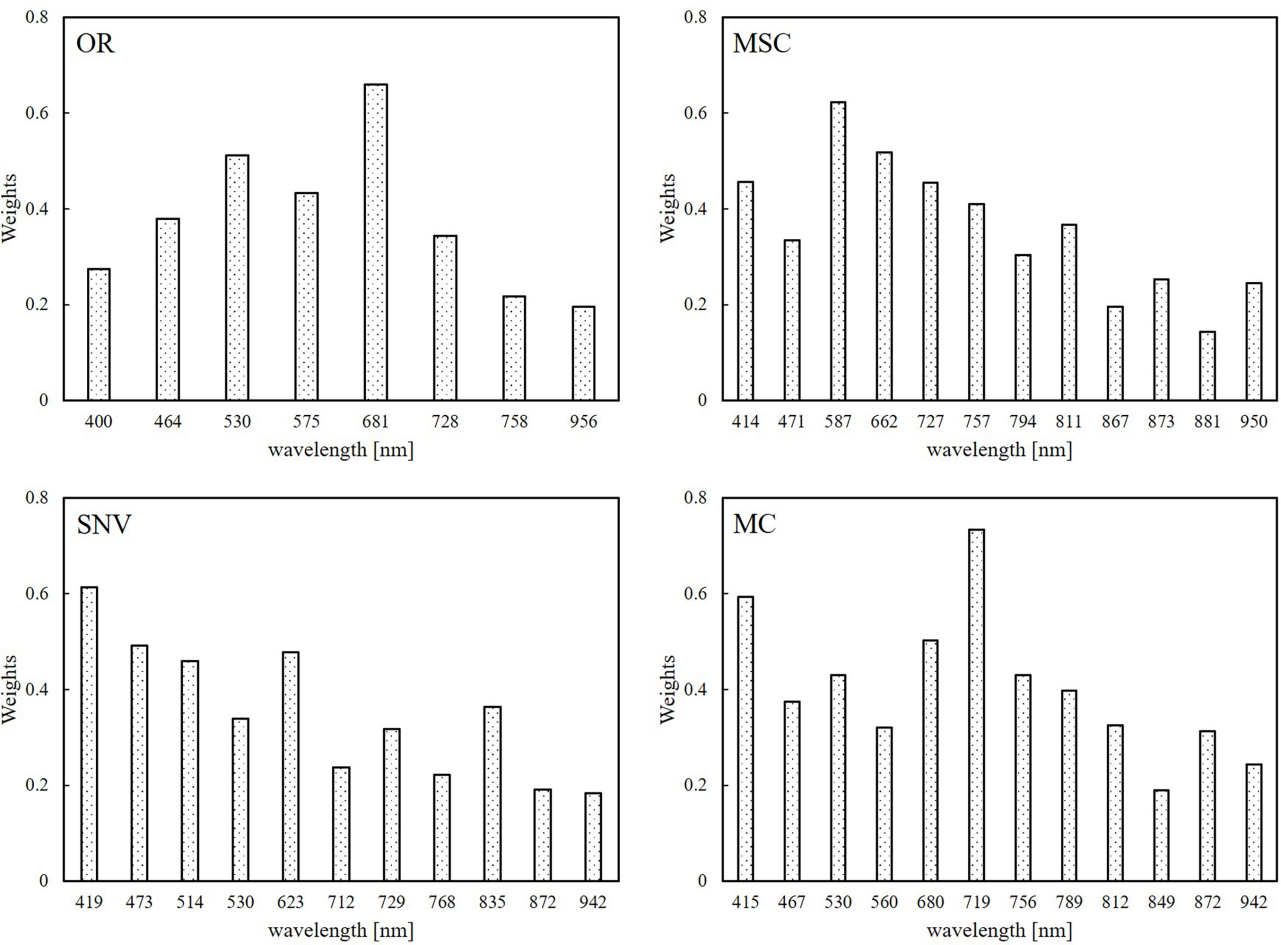
Feature band weights for the random forest regression (RFR) model based on four different spectral data transformation methods.

Random forest regression can calculate the importance of a single feature variable and sort by looking at the contribution rate of each feature in each decision tree, and the contribution rate uses the Gini coefficient as the evaluation index. Regardless of the spectral transformation data, the established RFR models performed best. The importance of the input variables for each model is shown in Figure 9. On the whole, the weight of the visible light band under different spectral transformations was higher than that of the near-infrared band; in particular, the weight ratio of red and green light was larger, while the weight of the near-infrared band was smaller. This is related to the obvious response of the plant pigments in wheat plants infected by the pathogen. At the same time, the disease affected the plant pigments before the canopy structure, which also led to the lower weight of the near-infrared bands estimated by the model. For the better-performing SNV-RFR and MC-RFR models, the weight of the blue band was further strengthened. As far as the best MC-RFR model is concerned, the bands with higher weights are mainly red edge, blue light, and red light.

## Discussion

Previous studies have confirmed the importance and application value of reflectance spectrum data in crop disease monitoring from different scales. It has been clearly established that the visible light and near-infrared regions are the sensitive bands for the identification of different crop diseases, but the spectral sensitivity bands of different crops and different types of damage are not the same (Xie et al., 2018). The sensitive band for wheat powdery mildew is mainly located at 580–720 nm (Graeff et al., 2006). Based on the spectral characteristics of the canopy, a new vegetation index for wheat powdery mildew, NDVI_1_ (Huang et al., 2019b), was constructed, and a good estimate effect was obtained. Recently, He et al. (2020) analyzed the appropriate monitoring angles of wheat powdery mildew from different observation angles and established a new vegetation index, RPMI, which has expanded the application scope of the monitoring model. Previous researchers constructed suitable vegetation indices by screening sensitive bands, but most of them only monitored diseases through two-band or three-band combinations. However, the waveband information used is relatively small and the inversion results have much room left for improvement. As we all know, hyperspectral remote sensing contains a lot of information, and the occurrence of diseases gradually develops from internal physiology and biochemistry to external morphological structure, and then shows external features that can be detected by remote sensing. Mesophyll cells, water, chlorophyll, nitrogen content, and leaf area index showed different characteristics, thus indicating the unique spectral properties at the different bands (Feng et al., 2019). Therefore, the use of multivariate analysis methods to accurately monitor diseases has become a popular topic in quantitative remote sensing research. The pathogenesis and mechanism of different diseases are not the same, especially for wheat powdery mildew, the pathogenesis characteristics are more special. The incidence of powdery mildew is from bottom to top, the early disease features are concealed, and the canopy spectrum is affected by plant structure, planting density, and stage. Therefore, the machine learning algorithm could be employed to coordinate multivariate factors well, thereby enhancing the covariant relationship between the canopy spectrum information and the disease index.

Spectral preprocessing methods could eliminate spectral noise and scattering effects and then enhance spectral characteristics that have been fully confirmed and widely used by previous studies. Gao et al. (2019) used the first derivative, the continuous removal method, and logarithmic transformation to perform spectral preprocessing when estimating the phosphorus content of plateau pastures, which showed that the first derivative is the best preprocessing method. Zhang et al. (2019b) compared different spectral pretreatment methods when testing wheat grain vigor, and found that MC was the best processing method. Liang et al. (2020) considered MSC and SNV to be the best methods to identify wheat grains infected with head blight and detecting deoxynivalenol (DON) content in wheat flour. Previous studies have proven the effectiveness of spectral preprocessing methods in different crops and different indexes, but some methods have changed the shape of the spectral curve while preprocessing the spectrum. Therefore, this study used the preprocessing methods that did not change the shape of the spectral curve, and thoroughly explored the applicability of the MC, MSC, and SNV methods in the monitoring of wheat powdery mildew. By comparing correlation coefficients, conventional vegetation indexes, and characteristic bands combined with machine learning modeling methods, generally speaking, when using the preprocessing method that does not change the spectral curve, MC was the best solution and most in line with the change law of the original spectral reflectance, and SNV transformation could also be used as a good preprocessing method.

After proper preprocessing of the spectral data, the selection of characteristic bands is very important. With the rapid development of computational methods, there are many methods for selecting characteristic bands. However, the characteristic bands used in different studies are very different. Zhao et al. (2020) found that when monitoring wheat powdery mildew at the leaf scale, the wavebands selected by the SPA algorithm were 423, 528, 597, 602, 645, 675, 714, 737, 774, and 1,057 nm, and the main wavebands were between 500 and 760 nm. Pane et al. (2021) used the random forest algorithm to reduce the dimensionality when monitoring *Diplotaxis tenuifolia* (Brassicaceae) infected with powdery mildew, and found that the important band related to powdery mildew was between 403 and 446 nm, and selected 403, 408, 411, and 446 nm as the characteristic wavebands. Yuan et al. (2014) compared the spectra of different diseases on winter wheat leaves, and determined that 666–685 nm, 752–758 nm, and 1,893–1,905 nm were the common sensitive bands for powdery mildew, stripe rust, and aphids, respectively. Therefore, it is particularly important to use the correct feature band selection method. In previous studies, the CARS algorithm was able to effectively extract the main spectral information from the whole band, but the CARS algorithm did not take into account the problem of multicollinearity and extracted more similar bands. The SPA algorithm selects characteristic variables by calculating the size of the projection vector of the remaining variables and the selected variables, which can ensure that the linear relationship between the selected variables is minimized, but the computational efficiency is often low. Based on this reality, this study used the CARS algorithm and the SPA algorithm together, which was able to extract the effective bands with low multicollinearity and improve the computational efficiency (Zhang et al., 2021). In this study, different spectral preprocessing methods were used, and the bands screened by the CARS–SPA algorithm were mainly distributed in the blue-green and red light ranges, and some near-infrared bands were also selected. The selected bands were distributed more uniformly, and the effect of blue light was enhanced, the weight of the red light was reduced, and the weight of the near-infrared band was the lowest. Therefore, the sensitive waveband screening method established in this study provides a reference for monitoring research of other related plant diseases.

With the popularization and updating of computer technology and the rapid development of agricultural information processing methods, the analyses represented by machine learning have greatly improved the correct identification of disease types, increased the accuracy of disease damage monitoring, allowing the rational application of pesticides to effectively avoid serious outbreaks of diseases and pests and ensure healthy crops and stable agricultural production. Machine learning algorithms can be used to effectively analyze and utilize information-rich data sets and high-dimensional observation data, and have been widely used in remote sensing data analysis and disease modeling and inversion (Han and Deng, 2018; Zheng et al., 2018). However, the machine learning algorithms selected for different crops and disease types are different, and the performance of different machine learning methods varies even under the same disease conditions (Chan et al., 2020). Jiang et al. (2021) demonstrated the good estimation ability of the RFR model in the study of mangrove diseases, and Zhang et al. (2020b) showed the superior classification performance of the RFR model in the identification of wheat grains infected with *Fusarium*. In this study, three modeling methods were used to establish an estimation model for the severity of wheat powdery mildew disease, and the RFR model performed best; this is mainly because the RFR algorithm has good anti-noise ability, is not easy to fall into over-fitting, and can solve most of the defects in the existing modeling methods (Meiforth et al., 2020). In general, by using the spectral data processed by MC and the 12 characteristic bands selected by the CARS–SPA algorithm, the established RFR model was shown to be the best model for estimating the disease index of wheat powdery mildew (*R*^2^ = 0.849–0.852). This indicates that the MC-CARS-SPA-RFR model is a good powdery mildew monitoring program. At the same time, the monitoring accuracy varies at different stages. The monitoring accuracy of flowering stage is better than that of filling stage, the reason for this being the relative stability of the canopy structure during flowering. While the canopy structure is more variable during the filling stage due to the senescence process, which reduces the stability of the monitoring. Different wheat varieties have different susceptibility to powdery mildew, which results in that the monitoring accuracy of the high-sensitivity types is higher than that of the medium-sensitivity types. When the severity of the disease is low, the complexity of the canopy structure makes the canopy spectrum contain less information about the lower plant, resulting in low accuracy of early monitoring. Monitoring the disease as early as possible will help prevent and control the disease as early as possible. The research on application requirements in this area still needs more researchers to pay more efforts to solve them.

In this study, the mDI was used to reduce the negative influence of the canopy structure, and the MC pretreatment method was employed to enhance the spectral characterization ability. Because the early symptoms of wheat powdery mildew disease are mainly concentrated in the lower part of the plant, it is difficult to fully extract the spectral signal from the lower part of the plant using a single inversion method, which in turn reduces the accuracy of disease monitoring. This article used a combination of several methods (MC-CARS-SPA-RFR) to enhance the extraction of the lower signal and its sensitivity to achieve ideal performance. The amount of resources required for this inversion mode is small, the calculation speed is fast, and the efficiency is high. Especially compared with CNNs, the advantages are more obvious. The optimal model for estimating powdery mildew established in this study showed that intelligent equipment can be used to monitor and analyze the degree of disease in real time, implement automatic perception, and accurately prevent and control disease in a timely manner, thereby promoting rapid and efficient agricultural production. Certainly, the methods in this paper are also used for practical application, evidently, a capital investment is initially required for adopting the employed approach (Taheri-Garavand et al., 2021b), as well as improvements and cost reductions to improve returns. In addition, remote sensing techniques at different scales have their own advantages. Ground-based remote sensing extracts the most realistic spectral information and is more suitable for accurate monitoring under small plots, UAV, and satellite remote sensing are respectively suitable for medium-scale farms and regional disease early warning. In crop-growing areas, where the plots are small and severely segmented, such as household planting farmers in China, the crop canopy spectrum can be measured with a handheld portable spectrometer to achieve precise field management of small plots. In addition, the results of this study also provide knowledge and technical support for the rapid and non-destructive monitoring of crop growth, as well as the real-time prevention and control of other types of diseases. Of course, the occurrence and characterization of powdery mildew disease is also related to the plant variety, growth period, and other environmental factors. It is also necessary to obtain more disease samples under different production environments and cultivation conditions for verification and improvement, so as to establish a more universal and applicable monitoring model. This will be of great significance and value for early prevention of crop diseases, improving prevention and control efficiency, and reducing yield loss.

## Conclusion

Under different spectral data preprocessing conditions, the application potential of the MC-CARS-SPA-RFR model in wheat powdery mildew monitoring was discussed and analyzed by combining characteristic band selection algorithms with machine learning. After comprehensive comparative analyses such as correlation, optimized combination of two bands, and machine learning modeling methods, MC was found to be the most effective spectral preprocessing method because it did not change the characteristics of the original spectral curve and significantly enhanced the correlation between it and the disease index. Based on the preprocessing of the spectral data, the CARS–SPA method was used to extract the characteristic bands. The number of bands extracted was greater than that of the original spectrum, and the band position distribution was more uniform. After the band selection using the MC-CARS-SPA method, different machine learning algorithms were used for regression modeling of powdery mildew disease severity, and thereby enhancing the covariant relationship between the canopy spectrum information and the disease index. The monitoring performance of the RFR model was found to be the best, and the monitoring accuracy of flowering stage is better than that of filling stage, the reason for this being the relative stability of the canopy structure during flowering. The weight of the visible light band in the selected characteristic bands was greater than that of near-infrared band, and the contributions of red edge, red light, and blue light were the largest. It can be seen that using the MC-CARS-SPA-RFR model algorithm enhanced the spectral response characteristics, extracted the characteristic bands more comprehensively and effectively, significantly improved the powdery mildew monitoring ability, and has a good prospect for application in the precise prevention and control of wheat powdery mildew. In order to further evaluate the robustness of the model, it needs to be tested and perfected under different crop types and environmental conditions.

## Data Availability Statement

The raw data supporting the conclusions of this article will be made available by the authors, without undue reservation.

## Author Contributions

XL, WF, and Z-HF conceived and designed the research. Z-HF, L-YW, and Z-QY analyzed the data and wrote the manuscript. XL, Y-YZ, LS, LH, and J-ZD provided data and data acquisition capacity. All authors contributed to the article and approved the submitted version.

## Funding

This work was supported by grants from the National Natural Science Foundation of China (31971791) and the Thirteenth Five-year Plan of National Key Research Project of China (2017YFD0300204).

## Conflict of Interest

The authors declare that the research was conducted in the absence of any commercial or financial relationships that could be construed as a potential conflict of interest.

## Publisher’s Note

All claims expressed in this article are solely those of the authors and do not necessarily represent those of their affiliated organizations, or those of the publisher, the editors and the reviewers. Any product that may be evaluated in this article, or claim that may be made by its manufacturer, is not guaranteed or endorsed by the publisher.
